# Amniotic Fluid Volume as a Contextual Marker of Latency and Perinatal Outcomes in Premature Prelabor Rupture of Membranes

**DOI:** 10.3390/jcm15135097

**Published:** 2026-06-30

**Authors:** Zoraya Mokachir-Mohsenin, Pilar López-Martínez, Javier Sánchez-Romero, José Eliseo Blanco-Carnero, Eva María Meroño-Saura, Elena Belando-Plaza, Elena Guillén-García, Romina Sol Liandro, Miriam Pertegal-Ruiz, Aníbal Nieto-Díaz, Catalina de Paco-Matallana

**Affiliations:** 1 Department of Obstetrics and Gynecology, ‘Virgen de la Arrixaca’ University Hospital, 30120 Murcia, Spain; z.mokachirmohsenin@um.es (Z.M.-M.); pili.lopezmart@gmail.com (P.L.-M.); eliblanco@um.es (J.E.B.-C.); evamaria.meronos@um.es (E.M.M.-S.); elenabelando14@gmail.com (E.B.-P.); elena.guillen3@carm.es (E.G.-G.); miriam.pertegal@um.es (M.P.-R.); anibal.nieto@um.es (A.N.-D.); 2 Department of Obstetrics and Gynecology, Pediatrics and Surgery, University of Murcia, 30120 Murcia, Spain; liandro.romina.sol@gmail.com; 3 Maternal-Fetal Medicine, Reproduction and Gynecology Research Group, Biomedical Research Institute of Murcia Pascual Parrilla–IMIB, 30120 Murcia, Spain; 4 Spanish Network in Maternal, Neonatal, Child and Developmental Health Research (RICORS-SAMID, RD24/0013/0018) Instituto de Salud Carlos III, 28040 Madrid, Spain

**Keywords:** premature rupture of fetal membranes, amniotic fluid, oligohydramnios, pregnancy outcome, gestational age

## Abstract

**Objectives**: The aim of this study was to evaluate the association between amniotic fluid volume at preterm prelabor rupture of membranes (PPROM) diagnosis and latency, short-term delivery risk, and perinatal outcomes across different gestational ages. **Methods**: This retrospective cohort study included singleton pregnancies with PPROM before 34 weeks’ gestation managed at a tertiary referral center. Amniotic fluid (AF) volume was categorized according to the deepest vertical pocket (≤20 mm vs. >20 mm). Fetal and neonatal outcomes were analyzed as predefined composite outcomes. Latency to delivery was assessed using Kaplan–Meier estimates. Multivariable Cox regression models were used to evaluate associations after adjustment for clinically relevant confounders. **Results**: A total of 263 pregnancies were included, of which 66.5% had an AF pocket ≤ 20 mm at presentation. Lower AF volume was associated with a higher incidence of the fetal composite outcome (74.3% vs. 52.3%, *p* < 0.001) and a higher short-term risk of delivery across gestational-age strata. In multivariable analysis, AF pocket > 20 mm remained independently associated with lower risk of fetal composite outcome. AF volume was not associated with overall latency to delivery (Spearman ρ = 0.03, *p* = 0.598). Although lower AF volume was associated with higher crude neonatal morbidity, this association was attenuated after adjustment for gestational age. **Conclusions**: Reduced AF volume at PPROM presentation was associated with adverse fetal outcomes and higher short-term delivery risk, whereas neonatal morbidity was mainly driven by gestational age at delivery. AF volume should therefore be interpreted as a contextual prognostic marker rather than an isolated determinant of outcome.

## 1. Introduction

Preterm prelabor rupture of membranes (PPROM) complicates approximately 3% of pregnancies and accounts for up to 30–40% of spontaneous preterm births, representing an important contributor to perinatal morbidity and mortality [1,2,3]. The condition is defined as rupture of the fetal membranes before 37 weeks of gestation and prior to the onset of labor. Management of PPROM requires balancing the risks of prematurity against complications associated with expectant management, including intraamniotic infection, placental abruption, and umbilical cord compression [2]. Neonatal complications commonly associated with PPROM include respiratory distress syndrome, bronchopulmonary dysplasia, early-onset sepsis, intraventricular hemorrhage, necrotizing enterocolitis, and long-term neurodevelopmental impairment, with gestational age at membrane rupture and delivery representing major determinants of neonatal outcome [1,4,5].

Amniotic fluid plays a critical role in fetal development, particularly in pulmonary maturation during mid-gestation [2,6]. Following membrane rupture, the loss of amniotic fluid may result in oligohydramnios, which has been associated with several adverse perinatal outcomes. Some studies have reported associations between oligohydramnios at PPROM diagnosis and adverse neonatal outcomes, including respiratory morbidity and neonatal death, although findings have been inconsistent across gestational ages and study populations [5]. In addition, oligohydramnios has been linked to shorter latency to delivery, higher rates of intraamniotic infection, and increased obstetric interventions [6,7].

Intraamniotic infection and inflammation are important components of the pathophysiology of PPROM and may further influence pregnancy outcomes. Clinical intraamniotic infection occurs in approximately 15–35% of cases and is more frequent at earlier gestational ages [2,8,9,10]. Several studies have examined the relationship between oligohydramnios, neonatal morbidity, and intraamniotic infection in pregnancies complicated by PPROM, although findings have been inconsistent [5,10,11]. Some authors have reported that oligohydramnios is independently associated with severe neonatal respiratory morbidity, pulmonary complications, and neonatal death, whereas others have found no significant association with neonatal outcomes after adjustment for gestational age and management strategy [10,11]. In addition, oligohydramnios has been associated with higher rates of positive amniotic fluid cultures, clinical and histological chorioamnionitis, and increased intraamniotic inflammatory activity [12], suggesting a complex interplay between residual amniotic fluid volume, infection, inflammation, and neonatal outcome that remains incompletely understood.

Despite previous studies suggesting that oligohydramnios may be associated with adverse outcomes in PPROM, important gaps remain in understanding the clinical significance of residual amniotic fluid volume after PPROM [5,7]. Most previous research has focused on respiratory morbidity in early PPROM or has evaluated heterogeneous neonatal outcomes, whereas the relationship between amniotic fluid volume and clinically relevant obstetric and perinatal outcomes has not been systematically assessed across a broad gestational age range in PPROM. The extent to which amniotic fluid volume provides clinically useful information beyond gestational age remains uncertain. In addition, whether residual amniotic fluid volume reflects different latency patterns or short-term delivery risk across gestational ages remains insufficiently explored.

The aim of the present study was to evaluate the association between amniotic fluid volume at PPROM diagnosis and latency, risk of imminent delivery, and perinatal outcomes according to gestational age at membrane rupture before 34 weeks. We hypothesized that reduced residual amniotic fluid volume at PPROM diagnosis would be associated with shorter latency, increased risk of imminent delivery, and worse perinatal outcomes, independently of gestational age.

## 2. Materials and Methods

This retrospective single-center cohort study was conducted at Hospital Clínico Universitario Virgen de la Arrixaca (Murcia, Spain). Singleton pregnancies complicated by PPROM before 34 weeks of gestation and managed at our institution between January 2009 and December 2023 were eligible for inclusion. Pregnancies with PPROM at or beyond 34 weeks were not included because institutional management protocols recommend delivery after diagnosis in these cases, precluding meaningful evaluation of latency and expectant management outcomes. Data were obtained from routinely collected clinical and ultrasound records.

The study was conducted in accordance with the principles of the Declaration of Helsinki. Ethical approval was obtained from the local institutional review board (2020-5-8-HCVUA), with a waiver of informed consent due to the retrospective design and anonymized nature of the dataset.

### 2.1. Participants

Eligible for inclusion were singleton pregnancies complicated by PPROM before 34 weeks’ gestation with known delivery and neonatal outcome. Pregnancies were excluded if they were affected by structural anomaly, chromosomal abnormality or PPROM before 16 weeks’ gestation. PPROM diagnosis was based on clinical assessment, including visualization of amniotic fluid pooling through the cervix during sterile speculum examination supported, when necessary, by ultrasound findings and biochemical testing according to institutional protocols. Amniotic fluid volume was assessed at PPROM diagnosis using the deepest vertical pocket (DVP) measured by ultrasound. Oligohydramnios was defined a priori as a deepest vertical pocket (DVP) ≤ 20 mm, according to ACOG and ISUOG recommendations [13,14].

Gestational age was determined by the measurement of crown–rump length (CRL) at 11–13 weeks [15]. Maternal characteristics, ultrasound findings, delivery data, and neonatal outcomes were extracted from the institutional ultrasound database (ViewPoint^®^, Webling, Germany) and electronic medical records.

Our center adheres to the NICE and local practice guidelines on the management of PPROM [16,17]. Termination of pregnancy was discussed in cases of PPROM before 23 weeks of gestation according to institutional policy and patient preference. When PPROM occurred after 23 weeks of gestation or when termination was rejected, patients were offered either inpatient expectant management or outpatient follow-up according to clinical stability and patient preference. Antibiotic therapy consisted of intravenous ampicillin and gentamicin followed by oral azithromycin according to institutional protocols. Clinical chorioamnionitis was defined by the presence of maternal fever associated with one or more additional clinical or laboratory findings, including maternal tachycardia, fetal tachycardia, uterine tenderness, foul-smelling vaginal discharge, or maternal leukocytosis [18].

Maternal clinical evaluation, laboratory testing, fetal ultrasound, and cardiotocography were routinely performed twice weekly during expectant management. Patients underwent expectant management in the absence of clinical signs of chorioamnionitis or other maternal or fetal contraindications. In the absence of maternal or fetal contraindications, expectant management was continued until 34 weeks of gestation, although timing of delivery was individualized in selected cases. Antenatal corticosteroids (betamethasone 12 mg intramuscularly every 24 h for two doses) were administered when delivery before 34 + 6 weeks was considered likely [19]. Magnesium sulfate for fetal neuroprotection was administered when delivery before 32 weeks was considered imminent [19].

### 2.2. Statistical Analysis

Gestational age at PPROM was categorized into four clinically relevant groups: <20 weeks, 20–25 weeks, 25–30 weeks, and 30–34 weeks. Missing data were handled using a complete-case approach. No imputation procedures were performed. Pregnancies with missing amniotic fluid measurements at PPROM diagnosis and/or missing outcome were excluded because amniotic fluid volume constituted the primary exposure variable.

Because isolated adverse events were infrequent, composite outcomes were predefined to improve statistical stability; individual outcome components were also analyzed separately. Fetal composite adverse outcome was considered in case of a stillbirth or iatrogenic early PTB (delivery < 34 weeks). Iatrogenic delivery before 34 weeks was included in the fetal composite because it reflects clinically significant fetal or maternal deterioration prompting interruption of expectant management. Neonatal composite adverse outcome was considered in cases where a newborn required CPAP or invasive mechanical ventilation in cases of respiratory distress syndrome, grade III or IV intraventricular hemorrhage, necrotizing enterocolitis, sepsis or retinopathy, or if a newborn developed anemia requiring blood transfusion. Neonatal sepsis was defined as culture-proven infection requiring antibiotic treatment. Necrotizing enterocolitis and intraventricular hemorrhage were classified according to standard neonatal diagnostic criteria [18].

Continuous variables were summarized as mean ± standard deviation or median (interquartile range) and were compared using Student’s *t*-test or Mann–Whitney U test, as appropriate. Categorical variables were summarized as counts and percentages and compared using the chi-square test.

Latency duration was described using Kaplan–Meier estimates. Median latency times and 95% confidence intervals were calculated for each gestational age group [20] and graphically represented using point estimates with horizontal confidence intervals. Time-to-event analyses were considered appropriate because most neonatal adverse outcomes occurred during the first hours or days after birth, allowing assessment of the temporal relationship between delivery and neonatal morbidity.

Delivery within 7 days after PPROM was analyzed as a short-term latency outcome. The risk of delivery within 7 days was calculated within each gestational-age stratum and amniotic-fluid-pocket category and expressed as a percentage.

Multivariable Cox regression models were constructed to evaluate the association between AF pocket and fetal and neonatal composite outcomes. For neonatal outcomes, time-to-event was calculated from birth to occurrence of the first adverse neonatal event or hospital discharge. Multivariable models were adjusted for clinically relevant confounders identified a priori, including gestational age at PPROM, maternal age, parity, and birthweight when appropriate. Model assumptions were assessed using standard diagnostic procedures, including visual inspection of residuals and assessment of model fit.

A *p*-value < 0.05 was considered statistically significant. All statistical analyses were performed using R statistical software (version 4.3.0, R Core Team, 2023) and Stata/BE 19.5 (StataCorp, College Station, TX, USA).

Generative artificial intelligence (ChatGPT, OpenAI, San Francisco, CA, USA; GPT-5.5, 2026) was used exclusively for English-language editing and improvement of manuscript readability. All scientific content, study design, statistical analyses, interpretation of results, and final manuscript revisions were performed and approved by the authors.

## 3. Results

### 3.1. Study Population and Baseline Characteristics

**Figure 1:**
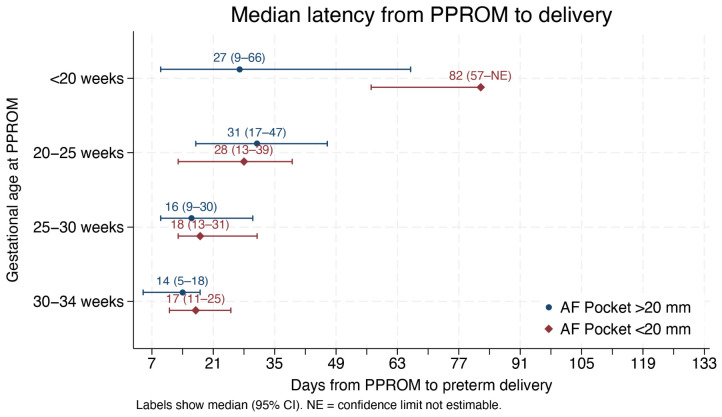
Median latency from PPROM to delivery according to gestational age at membrane rupture and amniotic fluid volume.

A total of 289 pregnancies complicated by preterm premature rupture of membranes (PPROM) were initially identified (Figure 1). Among the 289 identified pregnancies, 10 ended in termination of pregnancy. These cases were retained for descriptive analyses of latency and pregnancy course but excluded from analyses of perinatal outcomes. An additional 16 pregnancies (5.5%) were excluded because information on amniotic fluid pocket at PPROM diagnosis was unavailable. These cases were excluded from the analysis because amniotic fluid volume constituted the primary exposure variable. Therefore, 263 pregnancies with known perinatal outcomes were included in the final outcome analyses. Overall, 221 (84.0%) resulted in live births, 25 (9.5%) in intrauterine fetal demise, and 17 (6.5%) in neonatal death.

Baseline characteristics are summarized in Table 1. The median gestational age at PPROM was 28.1 weeks (interquartile range [IQR] 23.6–31.7). At presentation, 175 pregnancies (66.5%) had an amniotic fluid (AF) pocket ≤ 20 mm, and 88 (33.5%) had a pocket > 20 mm.

Baseline maternal characteristics, including maternal age, body mass index, parity, and conception method, were similar between groups. Pregnancies with AF pocket > 20 mm presented at earlier gestational ages and had a higher gestational age at delivery and birthweight, likely reflecting differences in gestational age distribution between groups. Pregnancies with a lower amniotic fluid volume delivered at earlier gestational ages and had lower birthweight neonates.

### 3.2. Latency from PPROM to Delivery

The median latency from PPROM to delivery was 15 days (interquartile range [IQR] 7–32). The distribution of latency times is shown in Figure 1.

Median latency differed across gestational age categories at PPROM. Pregnancies with PPROM before 20 weeks showed the longest latency intervals, whereas shorter latency periods were observed in pregnancies with PPROM between 30 and 34 weeks (Figure 1 and Figure S1).

**Figure 2:**
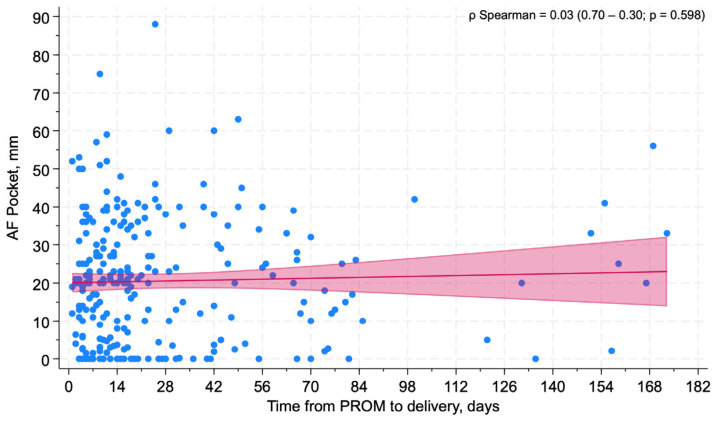
Relationship between amniotic fluid volume at presentation and latency to delivery in pregnancies with PPROM.

**Figure 3:**
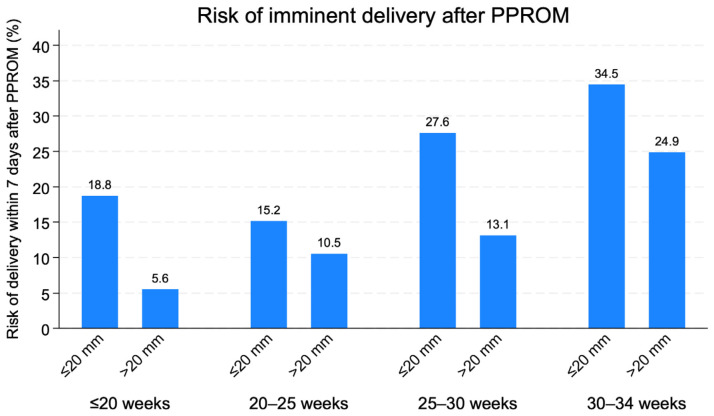
Probability of delivery within 7 days after PPROM according to amniotic fluid volume at presentation.

Although AF pocket size was not significantly correlated with overall latency duration (Figure 2 and Spearman ρ = 0.03, *p* = 0.598), lower AF volume was consistently associated with a higher risk of delivery within 7 days across all gestational-age strata (Figure 3). Median latency intervals showed substantial overlap between AF groups across gestational age strata (Figure 1). When amniotic fluid volume was modeled as a continuous variable, the predicted probability of delivery within 7 days decreased progressively with increasing pocket size, without evidence of a clear threshold effect at 20 mm (Figure S1).

### 3.3. Fetal Composite Outcome

**Figure 4:**
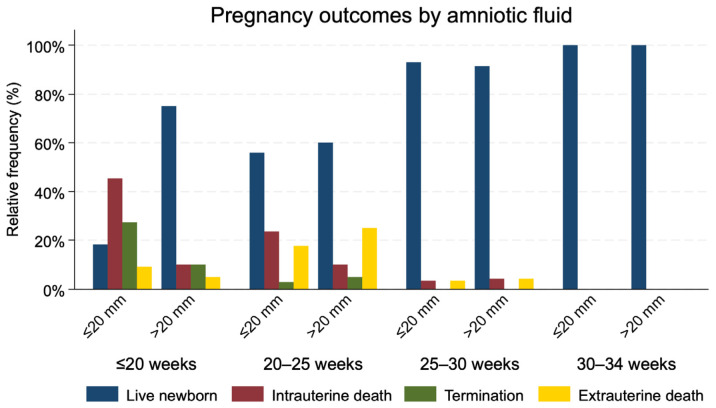
Pregnancy outcomes according to gestational age at PPROM and amniotic fluid volume at presentation.

**Figure 5:**
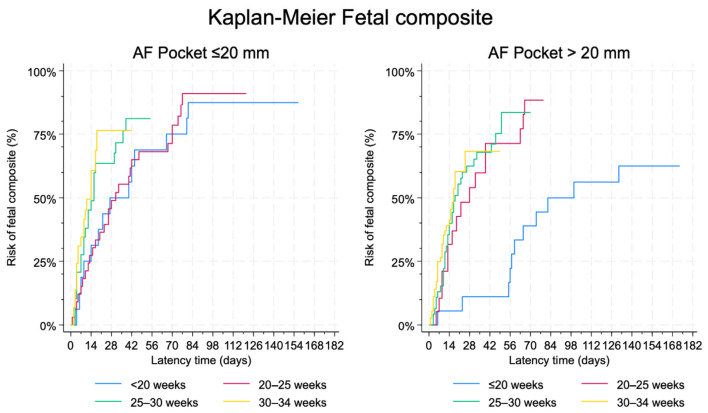
Cumulative incidence of the fetal composite outcome according to gestational age at PPROM and amniotic fluid volume.

Perinatal outcomes are summarized in Figure 4 and Table 2. The cumulative incidence of the fetal composite outcome according to gestational age at PPROM and latency is shown in Figure 5.

The fetal composite outcome occurred in 130 (74.3%) pregnancies with AF pocket ≤ 20 mm and in 46 (52.3%) pregnancies with AF pocket > 20 mm (*p* < 0.001).

In multivariable analysis, AF pocket > 20 mm was independently associated with a lower risk of fetal composite outcome (HR 0.6, 95% CI 0.4–0.8; *p* = 0.001). When stratified by amniotic fluid volume, pregnancies with an amniotic fluid pocket ≤ 20 mm showed a higher cumulative incidence of the fetal composite outcome compared with those with an amniotic fluid pocket > 20 mm (Figure 5).

The observed risk of delivery within 7 days after PPROM was consistently higher in pregnancies with AF pocket ≤ 20 mm across all gestational-age strata, particularly among pregnancies with PPROM between 25 and 34 weeks (Figure 3).

A multivariable Cox proportional hazards model evaluating factors associated with the fetal composite outcome is shown in Table 3.

### 3.4. Neonatal Composite Outcome

**Figure 6:**
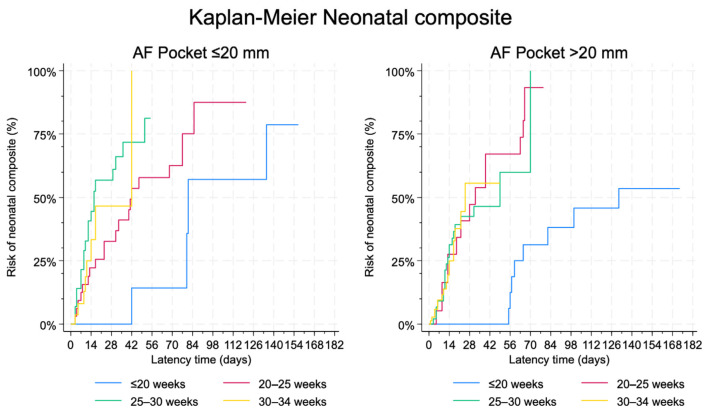
Cumulative incidence of the neonatal composite outcome according to gestational age at PPROM and amniotic fluid volume.

The neonatal composite outcome occurred in 79 (45.1%) pregnancies with AF pocket ≤ 20 mm and 31 (35.2%) pregnancies with AF pocket > 20 mm (*p* = 0.124). The cumulative incidence of the neonatal composite outcome according to gestational age at PPROM and latency is shown in Figure 6.

When stratified by amniotic fluid volume, pregnancies with an amniotic fluid pocket ≤ 20 mm showed a numerically higher cumulative incidence of the neonatal composite outcome compared with those with AF pocket > 20 mm (Figure 6).

The distribution of pregnancy outcomes according to gestational age at PPROM and amniotic fluid volume is presented in Figure 4. Higher proportions of intrauterine death and neonatal death occurred in pregnancies with earlier gestational age at membrane rupture and lower amniotic fluid pocket measurements.

A multivariable Cox proportional hazards model evaluating factors associated with the neonatal composite outcome is presented in Table 3. Most neonatal adverse events occurred within the first days after delivery.

## 4. Discussion

The present study examined the association between amniotic fluid volume at PPROM diagnosis and perinatal outcomes in pregnancies complicated by PPROM before 34 weeks. Pregnancies with an amniotic fluid pocket ≤ 20 mm showed a higher risk of the fetal composite outcome, including stillbirth and iatrogenic preterm delivery before 34 weeks. In contrast, amniotic fluid volume did not predict latency to delivery, suggesting that AF volume is not a major determinant of the timing of birth. Although pregnancies with lower AF volume showed higher crude rates of adverse neonatal outcomes, this association was attenuated after accounting for gestational age, suggesting that the association between AF volume and neonatal morbidity is largely mediated by gestational age at delivery.

Previous studies and current clinical guidelines support a relationship between reduced AF volume and adverse perinatal outcomes in PPROM. The Society for Maternal-Fetal Medicine identifies gestational age at PPROM and residual amniotic fluid volume as key factors associated with perinatal survival [4]. Oligohydramnios is independently associated with severe neonatal respiratory morbidity after adjustment for relevant confounders [5]. Our findings are consistent with previous studies and extend them across a broader gestational age range. Residual amniotic fluid volume after PPROM may reflect the severity of membrane disruption or the underlying inflammatory or placental process rather than acting as a direct causal determinant of neonatal morbidity [4,5].

AF volume did not appear to influence latency to delivery in our cohort. This differs from some reports suggesting shorter latency in the presence of oligohydramnios [7,21]. Other studies indicate that latency is influenced by multiple factors, including cervical characteristics and intrauterine inflammation, and that AF volume alone may have limited predictive value [22]. The heterogeneity across studies may reflect differences in inclusion criteria, gestational age at PPROM, definitions of oligohydramnios, and management protocols. Our findings suggest that, when evaluated across a wide gestational age spectrum, amniotic fluid volume at presentation does not independently determine latency duration.

The 20 mm threshold used to define oligohydramnios in this study should not be interpreted as an optimal prognostic cut-off. We selected this threshold a priori because it corresponds to the widely accepted clinical definition of oligohydramnios based on the deepest vertical pocket, as recommended by international guidelines [13,14]. Analyses modeling amniotic fluid volume as a continuous variable showed no meaningful correlation between DVP and overall latency duration, while demonstrating a gradual inverse relationship between AF volume and the predicted risk of delivery within 7 days. Importantly, no clear threshold effect was identified around the 20 mm value. These findings suggest that amniotic fluid volume may be better interpreted as a continuous marker of risk rather than as a dichotomous predictor defined by a single prognostic threshold.

Adequate amniotic fluid volume is essential for normal pulmonary maturation, particularly during mid-gestation [6]. Oligohydramnios reduces the protective fluid cushion surrounding the umbilical cord, increasing the risk of cord compression, fetal heart rate abnormalities, and fetal compromise requiring delivery [23,24]. Adequate amniotic fluid volume is essential for normal pulmonary development. Persistent oligohydramnios may impair airway distension and lung growth, increasing the risk of pulmonary hypoplasia and respiratory morbidity, especially when PPROM occurs at earlier gestational ages [11,25,26]. The risk of pulmonary hypoplasia decreases substantially after 23–24 weeks of gestation, highlighting the modifying role of gestational age [27,28,29]. Furthermore, intraamniotic infection and inflammation, which are frequently associated with both PPROM and reduced amniotic fluid volume, may contribute to fetal organ injury through inflammatory pathways and adverse pulmonary outcomes [30,31]. These mechanisms may act synergistically and could partly explain why the association between low amniotic fluid volume and adverse outcomes appeared more pronounced at earlier gestational ages in our cohort.

The relationship between AF volume and neonatal morbidity was considerably weaker. Although lower AF volume was associated with higher unadjusted rates of neonatal morbidity, this relationship appeared largely explained by gestational age at delivery. This finding reinforces the central role of prematurity as the dominant determinant of neonatal outcome in PPROM and suggests that the independent contribution of amniotic fluid volume to neonatal morbidity may be limited particularly after the threshold of fetal viability has been reached. These results are in line with previous studies indicating that gestational age at delivery is the strongest predictor of neonatal outcomes in this setting [4,9].

The prognostic relevance of amniotic fluid volume varied according to gestational age at PPROM rather than latency duration. In our study, the difference in outcomes between pregnancies with low and preserved amniotic fluid volume was more pronounced at earlier gestational ages, whereas this distinction became less evident at later gestational ages.

Residual amniotic fluid volume may have greater prognostic relevance in early PPROM, where severe oligohydramnios may reflect more extensive membrane disruption, prolonged fetal exposure to reduced fluid volume, or underlying intraamniotic pathology. In contrast, at later gestational ages, the overall prognosis is more strongly influenced by prematurity, which may attenuate the relative contribution of amniotic fluid volume.

Overall, these findings suggest that amniotic fluid volume may serve as a contextual indicator of baseline risk, although this interpretation should be made cautiously given the observational design and the influence of unmeasured factors such as intraamniotic infection or inflammation. Ultrasound markers, including oligohydramnios, have shown limited performance in predicting infectious complications or composite neonatal outcomes when used in isolation [32].

Further prospective multicenter studies are needed to validate these findings and to explore integrated predictive models combining clinical, ultrasound, and biochemical markers. The potential value of serial assessment of amniotic fluid volume during expectant management remains uncertain and warrants further investigation. Interventional approaches aimed at modifying amniotic fluid volume, such as amnioinfusion, require evaluation in well-designed studies to determine whether amniotic fluid volume represents a modifiable risk factor or primarily a prognostic indicator [33].

### Strengths and Limitations

Strengths of this study include the large population recruited for analysis and the use of standardized management protocols, predefined clinically relevant composite outcomes, and multivariable analysis adjusting for confounders. The inclusion of pregnancies across a broad gestational age range of PPROM before 34 weeks enhances the applicability of the findings to a broad clinical population.

Several limitations should be considered. The retrospective design may introduce selection bias and residual confounding. The exclusion of pregnancies with missing amniotic fluid data and those ending in termination of pregnancy could have introduced selection bias, although the number of such cases was relatively small. Although pregnancies ending in termination were excluded from outcome analyses and management was primarily guided by gestational age and overall clinical status, residual bias related to clinician assessment of disease severity cannot be completely excluded. No a priori sample size calculation was performed because this was a retrospective cohort study including all eligible cases during the study period. The study may have been underpowered to detect small differences for some relatively infrequent outcomes. The study was conducted over a prolonged recruitment period, during which changes in obstetric management, neonatal care, and viability thresholds may have occurred. Although institutional PPROM management protocols remained broadly consistent, temporal improvements in perinatal care could have influenced outcome rates and introduced unmeasured heterogeneity. The study did not include biomarkers of intraamniotic infection or inflammation, which may provide additional prognostic value. These factors may act as important confounders because they have been associated with both reduced amniotic fluid volume and adverse perinatal outcomes in PPROM. Unmeasured inflammatory processes may therefore have influenced the observed associations. The fetal composite outcome included iatrogenic delivery before 34 weeks, which may partially reflect clinical management decisions. Some of the observed associations between reduced amniotic fluid volume and adverse fetal outcomes could be influenced by indication bias rather than representing a direct biological effect of oligohydramnios. Amniotic fluid volume was assessed at a single time point and modeled as a fixed exposure, whereas it is inherently dynamic over time; this may have led to misclassification and potential underestimation of its association with outcomes. Finally, long-term neurodevelopmental outcomes were not evaluated, and these may be influenced by both gestational age and intrauterine conditions.

## 5. Conclusions

Amniotic fluid volume at presentation in PPROM was independently associated with adverse fetal outcomes and higher short-term risk of delivery, whereas its association with neonatal composite outcome was largely attenuated after adjustment for gestational age. These findings support the interpretation of amniotic fluid volume as a contextual prognostic marker whose clinical relevance varies according to gestational age and outcome definition.

## Figures and Tables

**Table 1:** Baseline maternal and pregnancy characteristics according to amniotic fluid volume at presentation in pregnancies with PPROM before 34 weeks. Continuous variables are summarized as mean (standard deviation) and categorical variables as count (percentage). BMI: Body Mass Index. ART: Assisted Reproductive Technique. PTB: Preterm birth. DM: Diabetes Mellitus. AF: Amniotic Fluid. PPROM: Preterm prelabor rupture of membranes. ACS: Antenatal corticosteroids.

Variable	AF Pocket ≤ 20 mm (*n* = 175)	AF Pocket > 20 mm (*n* = 88)	Total (*n* = 263)	*p*
Maternal age, years	32.5 (6.4)	32.7 (6.7)	32.6 (6.5)	0.824
Weight, Kg	70.2 (16.7)	67.5 (14.6)	69.3 (16.0)	0.271
Height, cm	163.3 (6.6)	162.1 (7.4)	162.9 (6.9)	0.243
BMI, Kg/m^2^	26.6 (6.1)	25.9 (5.4)	26.3 (5.9)	0.475
Mode of conception				
Spontaneous	146 (91.2%)	67 (84.8%)	213 (89.1%)	0.133
ART	14 (8.8%)	12 (15.2%)	26 (10.9%)	
PTB history	18 (11.0%)	12 (14.0%)	30 (12.0%)	0.491
Chronic hypertension	1 (0.6%)	2 (2.3%)	3 (1.2%)	0.234
Diabetes mellitus	12 (7.3%)	4 (4.7%)	16 (6.4%)	0.420
Nulliparous	78 (45.6%)	37 (42.5%)	115 (44.6%)	0.637
Smoke	32 (18.3%)	8 (9.1%)	40 (15.2%)	0.050
AF pocket, mm	11.2 (8.9)	39.1 (10.9)	20.5 (16.3)	<0.001
Gestational age at PPROM, weeks	29.2 (4.3)	25.0 (5.3)	27.0 (5.3)	<0.001
<20 weeks	25 (14.3%)	9 (10.2%)	34 (12.9%)	0.094
20–25 weeks	41 (23.4%)	11 (12.5%)	52 (19.8%)	
25–30 weeks	48 (27.4%)	28 (31.8%)	76 (28.9%)	
30–34 weeks	61 (34.9%)	40 (45.5%)	101 (38.4%)	
Time from PROM to delivery, days	26.2 (31.0)	29.9 (35.4)	27.5 (32.5)	0.383
ACS	152 (91.0%)	81 (95.3%)	233 (92.5%)	0.224
Gestational age at ACS, weeks	28.2 (3.5)	29.2 (3.5)	28.6 (3.5)	0.050
Gestational age at delivery, weeks	29.8 (4.6)	31.2 (4.5)	30.5 (4.6)	0.011
<24 weeks	22 (12.6%)	4 (4.5%)	26 (9.9%)	0.002
24–28 weeks	39 (22.3%)	9 (10.2%)	48 (18.3%)	
28–32 weeks	50 (28.6%)	24 (27.3%)	74 (28.1%)	
32–37 weeks	60 (34.3%)	44 (50.0%)	104 (39.5%)	
>37 weeks	4 (2.3%)	7 (8.0%)	11 (4.2%)	
Labour onset				
Spontaneous	99 (60.0%)	40 (46.0%)	139 (55.2%)	0.082
Induced	38 (23.0%)	30 (34.5%)	68 (27.0%)	
No labour onset	28 (17.0%)	17 (19.5%)	45 (17.9%)	
Delivery				
Spontaneous	90 (53.9%)	53 (60.9%)	143 (56.3%)	0.284
Cesarean section	77 (46.1%)	34 (39.1%)	111 (43.7%)	

**Table 2:** Fetal and neonatal outcomes according to amniotic fluid volume at presentation in pregnancies complicated by PPROM. Continuous variables are summarized as mean (standard deviation) and categorical variables as count (percentage). AF: Amniotic Fluid. PPROM: Preterm prelabor rupture of membranes. NICU: Neonatal Intensive Care Unit. IMV: Invasive Mechanical Ventilation. CPAP: Continuous Positive Airway Pressure. RDS: Respiratory Distress Syndrome. IVH: Intraventricular Hemorrhage.

Variable	AF Pocket ≤ 20 mm (*n* = 175)	AF Pocket > 20 mm (*n* = 88)	Total (*n* = 263)	*p*
Latency from PPROM to delivery, days	26.2 (31.0)	29.9 (35.4)	27.5 (32.5)	0.383
Outcome				
Live newborn	140 (80.0%)	81 (92.0%)	221 (84.0%)	0.042
Intrauterine death	21 (12.0%)	4 (4.5%)	25 (9.5%)	
Extrauterine death	14 (8.0%)	3 (3.4%)	17 (6.5%)	
Birthweight, g	1672.2 (606.5)	2030.6 (687.9)	1801.7 (658.7)	<0.001
Apgar score at first minute	7.2 (2.3)	8.3 (1.4)	7.6 (2.1)	<0.001
Apgar score at fifth minute	8.7 (1.9)	9.4 (1.1)	8.9 (1.7)	0.002
Apgar score at tenth minute	9.1 (1.6)	9.5 (0.9)	9.2 (1.4)	0.086
Arterial blood cord pH	7.3 (0.1)	7.3 (0.1)	7.3 (0.1)	0.661
Venous blood cord pH	7.4 (0.1)	7.4 (0.1)	7.4 (0.1)	0.918
Fetal composite	130 (74.3%)	46 (52.3%)	176 (66.9%)	<0.001
Neonatal composite	79 (45.1%)	31 (35.2%)	110 (41.8%)	0.124
Neonatal admission	75 (52.1%)	43 (51.2%)	118 (51.8%)	0.896
NICU admission	68 (47.2%)	25 (29.8%)	93 (40.8%)	0.010
Ventilation	70 (51.5%)	28 (33.3%)	98 (44.5%)	0.009
IMV	39 (29.1%)	18 (22.0%)	57 (26.4%)	0.247
Time requiring IMV, days	3.5 (9.2)	1.0 (2.3)	2.7 (7.8)	0.091
Intubation	32 (23.7%)	12 (14.5%)	44 (20.2%)	0.099
Time requiring intubation, days	8.5 (10.9)	5.0 (4.1)	7.7 (9.9)	0.420
CPAP	47 (35.6%)	19 (23.2%)	66 (30.8%)	0.056
Time requiring CPAP, days	3.7 (6.9)	0.6 (1.2)	2.8 (6.0)	0.007
RDS	39 (29.8%)	19 (23.2%)	58 (27.2%)	0.292
Phototherapy	73 (54.5%)	43 (53.1%)	116 (54.0%)	0.843
Time requiring Phototherapy, days	1.8 (2.5)	1.4 (1.4)	1.6 (2.2)	0.306
IVH	11 (8.2%)	6 (7.1%)	17 (7.8%)	0.775
Grade 1	6 (54.5%)	4 (66.7%)	10 (58.8%)	0.231
Grade 2	4 (36.4%)	0 (0.0%)	4 (23.5%)	
Grade 3	0 (0.0%)	1 (16.7%)	1 (5.9%)	
Grade 4	1 (9.1%)	1 (16.7%)	2 (11.8%)	
Neonatal blood transfusion	11 (8.9%)	5 (6.3%)	16 (7.9%)	0.512
Necrotizing Enterocolitis	5 (3.7%)	5 (6.0%)	10 (4.6%)	0.427
Retinopathy	6 (4.6%)	3 (3.7%)	9 (4.2%)	0.758

**Table 3:** Multivariable Cox regression analysis of factors associated with the neonatal and fetal composite outcome in pregnancies with PPROM. HR: Hazard Ratio. CI: Confidence Interval. PPROM: Preterm prelabor rupture of membranes. AF: Amniotic Fluid. ACS: Antenatal Corticosteroids.

Fetal Composite	HR	CI95%	*p*
Gestational age at PPROM, weeks			
<20 weeks	Reference		
20–25 weeks	1.9	1.2–3.3	0.011
25–30 weeks	3.0	1.8–5.2	<0.001
30–34 weeks	3.9	2.3–6.9	<0.001
AF Pocket			
<20 mm	Reference		
>20 mm	0.6	0.4–0.8	0.001
Neonatal Composite	HR	CI 95%	*p*
Gestational age at PPROM, weeks			
<20 weeks	Reference		
20–25 weeks	7.2	3.0–16.9	<0.001
25–30 weeks	25.6	9.8–66.7	<0.001
30–34 weeks	125.9	37.7–420.6	<0.001
AF Pocket			
<20 mm	Reference		
>20 mm	0.9	0.6–1.5	0.784
ACS	1.5	0.3–6.2	0.598
Gestational age at delivery, weeks	1.0	1.0–1.0	<0.001

## Data Availability

The raw data supporting the conclusions of this article will be made available by the authors on request due to privacy and ethical restrictions related to the use of retrospective clinical patient data.

## Supplementary Materials

The following supporting information can be downloaded at: https://www.mdpi.com/article/10.3390/jcm15135097/s1, Figure S1: Predicted probability of delivery within 7 days according to amniotic fluid pocket at presentation in pregnancies with PPROM, stratified by pregnancy outcome.

## Abbreviations

The following abbreviations are used in this manuscript:

PPROM	Preterm prelabor rupture of membranes
AF	Amniotic fluid
NICU	Neonatal intensive care unit
IMV	Invasive mechanical ventilation
CPAP	Continuous positive airway pressure
RDS	Respiratory distress syndrome
IVH	Intraventricular hemorrhage
ACS	Antenatal corticosteroids
DVP	Deepest vertical pocket
