# Supplementary material for: Amniotic Fluid Volume as a Contextual Marker of Latency and Perinatal Outcomes in Premature Prelabor Rupture of Membranes
Source: J Clin Med. 2026 Jun 30;15(13):5097. doi: 10.3390/jcm15135097 (PMC13363472; doi:10.3390/jcm15135097)
Supplement: Supplementary file 1 [file jcm-15-05097-s001.zip › jcm-4363281-supplementary.pdf]

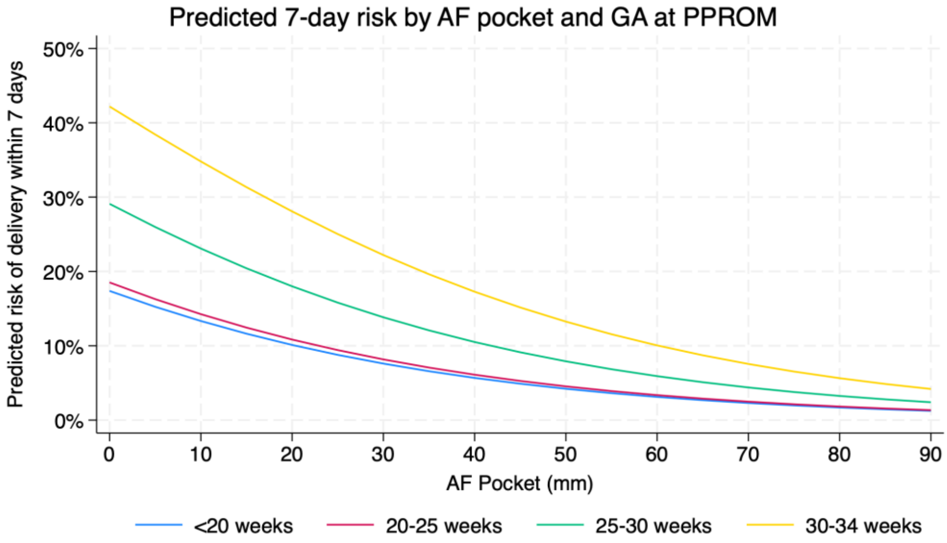

Figure S1. Predicted probability of delivery within 7 days according to amniotic fluid pocket at presentation in pregnancies with PPROM, stratified by pregnancy outcome.
